# The Long COVID experience from a patient's perspective: a clustering analysis of 27,216 Reddit posts

**DOI:** 10.3389/fpubh.2023.1227807

**Published:** 2023-08-17

**Authors:** Hanin Ayadi, Charline Bour, Aurélie Fischer, Mohammad Ghoniem, Guy Fagherazzi

**Affiliations:** ^1^Deep Digital Phenotyping Research Unit, Department of Precision Health, Luxembourg Institute of Health, Strassen, Luxembourg; ^2^Faculty of Science, Technology and Medicine, University of Luxembourg, Esch-sur-Alzette, Luxembourg; ^3^École doctorale Biologie, Santé, et Environnement, Université de Lorraine, Nancy, France; ^4^Luxembourg Institute of Science and Technology, Esch-sur-Alzette, Luxembourg

**Keywords:** public health, patient-reported outcomes, Long COVID, digital health, social media, artificial intelligence, natural language processing, machine learning

## Abstract

**Objective:**

This work aims to study the profiles of Long COVID from the perspective of the patients spontaneously sharing their experiences and symptoms on Reddit.

**Methods:**

We collected 27,216 posts shared between July 2020 and July 2022 on Long COVID-related Reddit forums. Natural language processing, clustering techniques and a Long COVID symptoms lexicon were used to extract the different symptoms and categories of symptoms and to study the co-occurrences and correlation between them.

**Results:**

More than 78% of the posts mentioned at least one Long COVID symptom. Fatigue (29.4%), pain (22%), clouded consciousness (19.1%), anxiety (17.7%) and headaches (15.6%) were the most prevalent symptoms. They also highly co-occurred with a variety of other symptoms (e.g., fever, sinonasal congestion). Different categories of symptoms were found: general (45.5%), neurological/ocular (42.9%), mental health/psychological/behavioral (35.2%), body pain/mobility (35.1%) and cardiorespiratory (31.2%). Posts focusing on other concerns of the community such as vaccine, recovery and relapse and, symptom triggers were detected.

**Conclusions:**

We demonstrated the benefits of leveraging large volumes of data from Reddit to characterize the heterogeneity of Long COVID profiles. General symptoms, particularly fatigue, have been reported to be the most prevalent and frequently co-occurred with other symptoms. Other concerns, such as vaccination and relapse following recovery, were also addressed by the Long COVID community.

## Introduction

As of November 2022, more than 630 million confirmed COVID-19 cases and 6.5 million consequential deaths have been reported by the WHO (1). Even though most infected people fully recover from the initial acute illness, at least 10–20% of the recovered patients experience mid and long-term effects after the said recovery (2).

Long COVID, officially referred to as Post-acute sequelae of SARS-CoV-2 infection (PASC), is a condition where people experience a variety of symptoms that persist from or develop after their initial infection with COVID-19 (3).

Long COVID symptoms are varied, multi-organ and can range from fatigue to cardio-respiratory issues, including cognitive dysfunction (4). These can interfere with the everyday life of affected individuals, preventing them from regular activities such as work and house chores (5).

Social media platforms played a fundamental role in the recognition of this condition, arguably considered the first patient-made illness through online campaigns (6). Reports of prolonged symptoms after infection were shared by patients on social media in the second half of 2020 and the term “Long COVID” was first adopted on Twitter by Elisa Perego through the hashtag “#LongCovid”. People who suffered from long-term effects of Covid-19 also referred to themselves as “long-haulers” (7).

Several support communities created by people suffering from different health conditions and diseases can be found online (8). Long-haulers built multiple ones on social media, including Reddit, to share their experiences with the illness, to understand their own condition, to find potential treatments and to provide support for one another (5).

The perception of Long COVID from a patient's position can differ from what is seen by non-affected individuals. Long-haulers especially requested further efforts from official health organizations, professionals and researchers to acknowledge the reality of their situation and to invest in research and treatment (9). In addition, patients tend to under-report their negative feelings and symptoms during appointments with healthcare professionals. This social desirability bias complicates the identification of Long COVID profiles (10).

In this context, conducting patient-centric research that focuses on people with Long COVID and what they share on social media platforms is essential. In fact, social media has been increasingly used for various health research purposes since 2009 (11, 12). It is a valuable and accessible source of information based on patient-reported outcomes, where most narratives around this new condition emerged and continue to grow. They also centralize the concerns and experiences of affected and neglected individuals on a global scale.

The use of data science and artificial intelligence (AI) methods contributed significantly to COVID-19 research (13). Similarly, using AI techniques to explore what people with PASC post online would help to uncover patterns and gain a deeper understanding of their unique symptomatic experiences associated with the condition.

Foufi et al. used text mining to extract and identify relationships between biomedical entities from self-reported outcomes shared by people with chronic diseases on Reddit (14). They emphasized the willingness of affected individuals to share their personal experiences with their illnesses online. This willingness is not limited to those with chronic illnesses as shown by Park and Conway's lexicon-based and supervised machine learning approaches, which confirmed the prevalence of communicative diseases discussions and public health concerns on Reddit (15). Long COVID was explored by Sarker and Ge through the Reddit community “covidlonghaulers” (16). Outside of social media, Long COVID research on health records was conducted. Wang et al. employed language processing tools to build a symptoms lexicon for Long COVID from clinical records (17) while Zhang et al. studied the characteristics of the condition and identified potential subphenotypes using machine learning (18).

Within this framework, this research work focused on analyzing self-reported Long COVID experiences on Reddit to identify potential profiles of the condition, not only in terms of individual symptoms but also the categories of symptoms and the eventual associations between them. We combined data analysis, lexicon-based and machine learning approaches to process text collected on Long COVID-related Reddit forums, then automatically extracted patterns relevant to the PASC condition.

## Materials and methods

In this section, we develop the different steps to collect, process and transform the data. The implementation of the various phases of the project can be found on this Github repository: HaninAyadi/DOVA_LUX (github.com).

**Figure 1:**
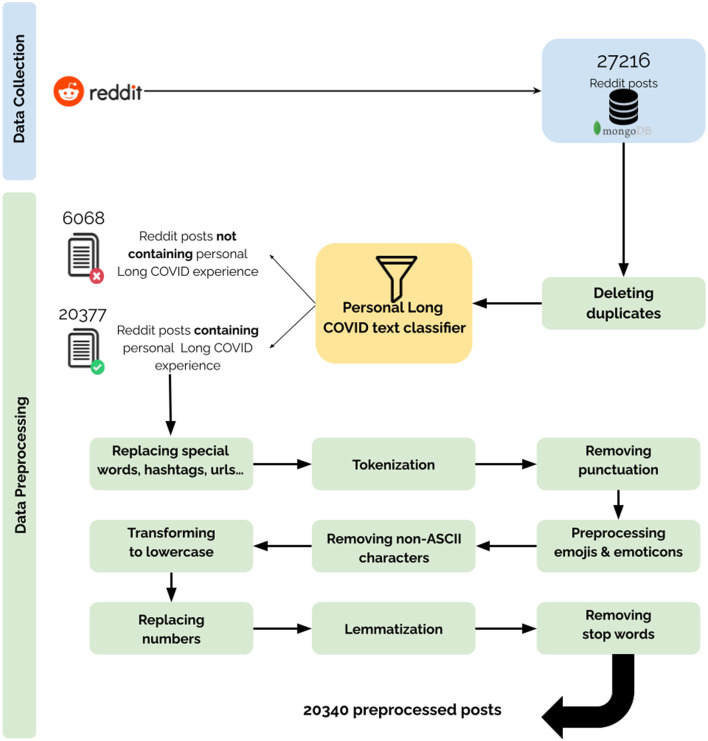
Data collection and preprocessing pipeline. Description of the performed steps for the collection and preprocessing of the data.

Figure 1 shows the Reddit data preparation stages before applying the clustering algorithm, which will be detailed in the following sections.

### Data collection

Reddit was chosen for the data collection for several reasons. First, data is publicly available, easy and free to collect using the Pushshift Application Programming Interface (API). This API was designed to provide enhanced functionality and search capabilities to search through Reddit comments and submissions, which facilitated the task of data collection (19). Second, Reddit allows posting long content which serves for a better elaboration of the patient experience. Third, during the data collection process, there is no limit to how far back in time you can go. Moreover, it is possible to collect the entire data from the forums at once without needing to set up continuous data collection processes.

The public Reddit forums or communities, also called subreddits, allow pseudonymous Reddit users to share posts, discuss and stay updated on a specific topic of interest while respecting community rules that are supervised by moderators. The existence of those dedicated monitored subreddits reduces the irrelevant content during data collection. The Long COVID subreddits we used are displayed in Supplementary material 1 with their respective number of collected posts and estimated number of members.

The final collection includes all posts since the creation of the forums along with descriptive metadata for each post, such as its id, title, creation timestamp, the pseudonym of the author and information about the subreddit (e.g., name, subscribers). The comments on each post were not collected.

### Data analysis

**Figure 2:**
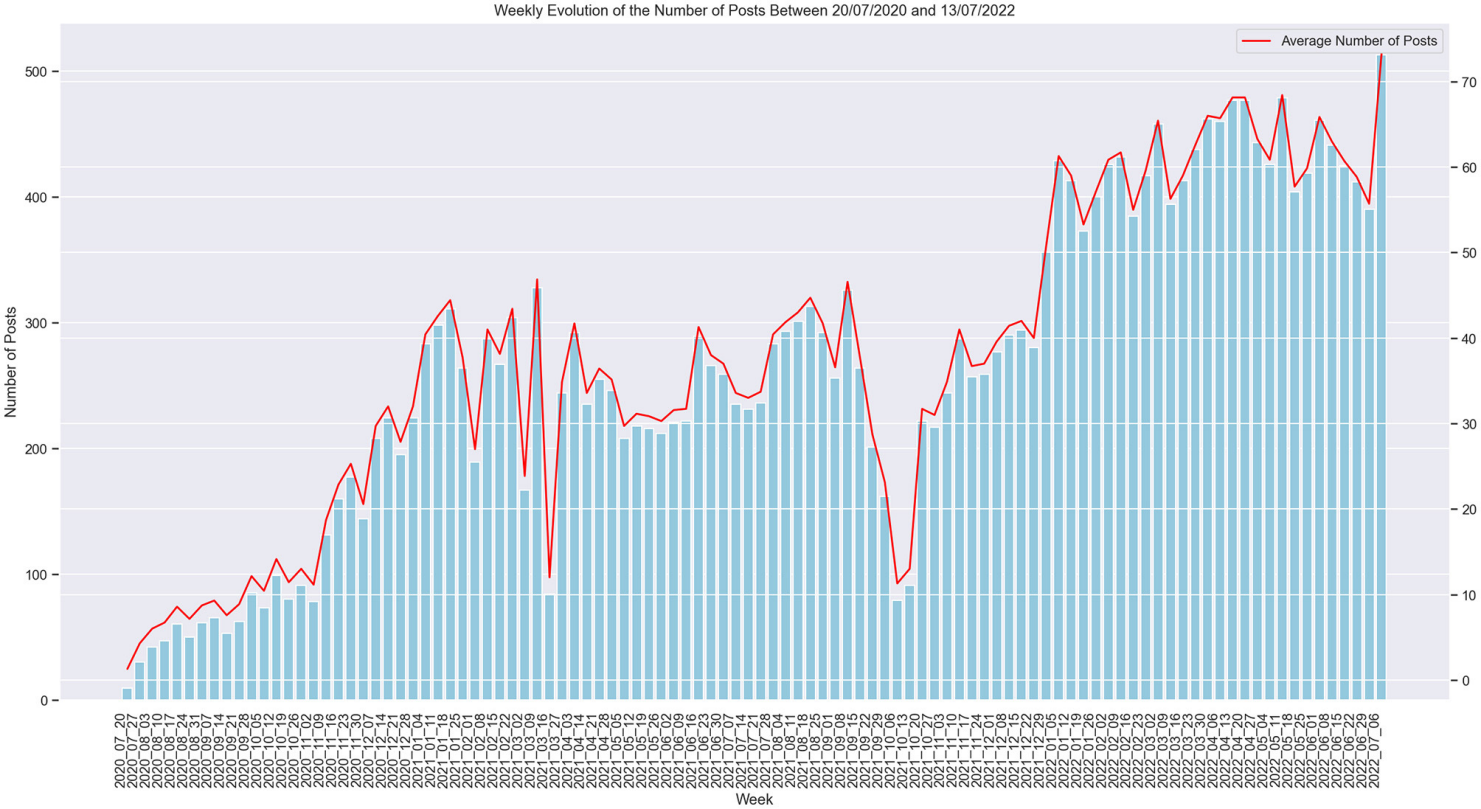
Weekly evolution of the number of posts.

Most of the posts were originally published in the “covidlonghaulers” subreddit, being the most popular Long COVID community. The raw dataset encompasses 27,216 posts from 8,434 distinct users with an average number of 3 posts per user (SD = 6, Q1 and Q2 = 1, Q3 = 3). The weekly evolution of the general number of posts over the span of 2 years can be seen in Figure 2. We inspected the length of the posts and found that the average number of words per post rounds up to 137 words (SD = 191, Q1 = 38, Q2 = 84, Q3 = 165). The longest post contained 5469 words while the shortest was only one word (which was then excluded during the preprocessing phase).

In the subsequent stages of this study, the statistical examination of symptoms, clusters and their associations involves the utilization of quantifiable measures such as percentages, frequencies and co-occurrence analysis.

### Personal/non-personal Long COVID text classifier

It is common that text discussing Long COVID can also include information about the acute infection with COVID-19 as well as news, scientific articles, or anything not descriptive of the personal experience itself with the long-term condition. As our goal was to investigate the experiences and perspectives of people with PASC, we needed to remove portions of the text that were not related to these aspects. For this particular purpose, we built a classifier that identifies sentences with explicit mentions of personally having or suspecting to have Long COVID or being close to someone who does, e.g., a family member.

Two authors (HA, CB) manually labeled a dataset containing a total of 1,395 text samples. This dataset consisted of random sentences from posts. In all, 402 were annotated as related to personal PASC experiences. Complicated cases were discussed between the two authors to obtain a 100% inter-rater agreement.

We then performed several preprocessing steps such as normalization to lowercase, expanding contractions and processing emojis and emoticons on the labeled samples. The 1,395 text examples were split into 1,116 training samples and 279 validation samples. We used BERTweet (20), a pre-trained language model based on RoBERTa (21), which stands for Robustly Optimized BERT Pre-training Approach. Its objective is to optimize the training of the BERT (22) architecture to reduce model pre-training time. Supplementary material 2 shows the classifier performance metrics.

### Data preprocessing

Multiple preprocessing steps were applied to the collected 27,216 English posts. First, we computed the pairwise cosine similarity between the Term Frequency-Inverse Document Frequency (TF-IDF) vectors of the Reddit posts to detect content-based duplicates. Second, the Personal/Non-personal Long COVID text classifier filtered out irrelevant posts and sentences. In fact, for each post, the sentences including an experience with Long COVID were kept. We ended up identifying 20,377 relevant posts. Third, we moved on to a standard preprocessing pipeline of replacing context-specific words and contractions, tokenization, removing punctuation, preprocessing emojis and emoticons, removal of non-ASCII characters, lowercasing and lemmatization.

Stop words are commonly used words such as the words “the” or “and” in English. To fit our context, we manually created a list of context-specific stop words. Amongst the 500 words with the lowest inverse document frequency score in our corpus, we selected those that were unimportant for identifying specific Long COVID patient profiles. Inverse document frequency measures how common a word is in a set of text samples. It is mathematically defined as the logarithm of the quotient: the total number of samples divided by the number of samples where the term is used. The output posts of the preprocessing pipeline are transformed into vectors using the TF-IDF weights.

### K-means clustering

K-means is an unsupervised clustering algorithm. A known challenge with K-means is the choice of K, the initial value of the number of clusters. We experimented with values ranging from 2 to 200 to then closely focus on a smaller interval and evaluate the results for each value. Several criteria were taken into consideration to validate the final clustering result. First, the silhouette score was computed based on the average distances between each data sample, the samples of the same cluster and those of the nearest neighboring cluster. This score is commonly used as an evaluation metric to assess how well the text sample fits in its current cluster compared to the other clusters (23). Second, the distribution of the posts across the clusters was also considered. For instance, obtaining multiple clusters containing a very low number of posts or obtaining a few enormous clusters both indicate that more values of K should be explored. Finally, the number of extracted symptom groups and their epidemiological interpretation also played an important role in finding the best number of clusters.

The Elbow method was used to approach the value of the number of clusters K. To label the clusters, we read at least the 20 closest text posts to each cluster center and looked at the most frequently used words and word pairs in the clusters.

We used the Python Scikit-learn library's implementation of Mini-Batch K-Means clustering which accelerates the learning for large-scale data. It does incremental updates of the cluster centers' positions using mini-batches of the samples instead of the whole set (24).

In addition, we used the t-distributed stochastic neighbor embedding (t-SNE) method for the projection of the high-dimensional TF-IDF input data vectors in a two-dimensional space (25). This visualization algorithm allows the discovery of the underlying structure in the data while preserving the similarities found between data points before transitioning to the lower dimensions. The visualizations were considered to assess and understand the results of K-means clustering for different values of K.

Finally, grouping the resulting clusters helped to facilitate the visualization and the assessment of the clustering. The labels used for the grouped symptom clusters referred to the predominant symptom category for each group.

### Symptoms lexicon

A symptom can be referred to by the posters using synonyms, acronyms, or different expressions. Thus, simply recognizing symptoms from the most frequently used words in the posts or each cluster does not allow an accurate identification of symptoms. Wang et al. (17) developed “PASClex”, a PASC symptom lexicon derived from 328,879 health record clinical notes of 26,117 COVID-19 positive patients in their post-acute infection period. It contains a total of 16,466 synonyms and expressions mapped to 355 different symptoms that are associated with Long COVID. We searched those words in our text data to establish a more accurate occurrence count for each symptom.

### Categorization of the symptoms

We mapped the 355 “PASClex” symptoms to 13 different categories that we identified according to the affected organs and functions. These categories can be seen in Supplementary material 3.

## Results

### Symptoms extraction

**Figure 3:**
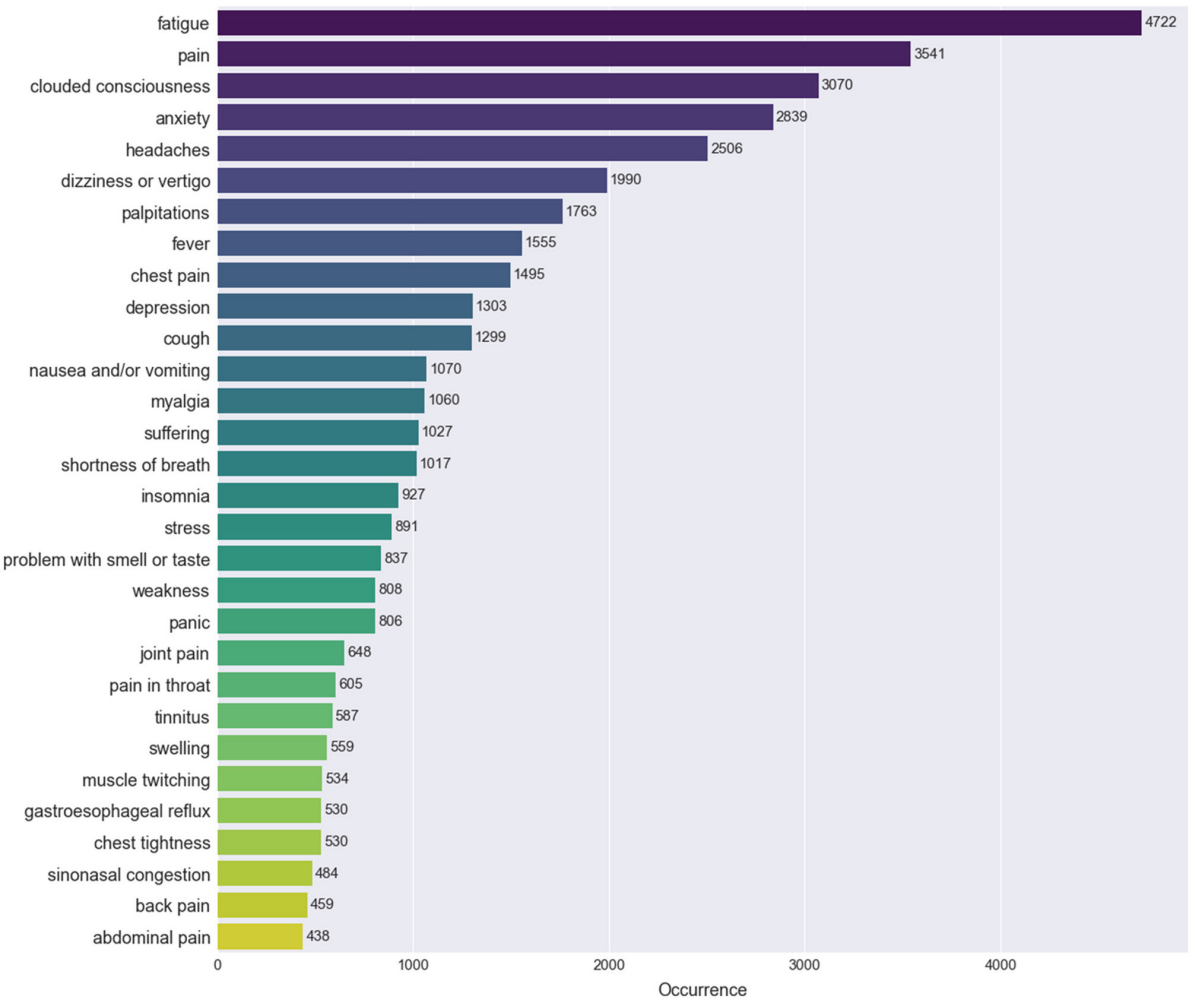
Most frequently extracted Long COVID symptoms. The 30 most frequently mentioned symptoms across the Reddit posts. The occurrence is the number of posts where the symptom was reported.

**Figure 4:**
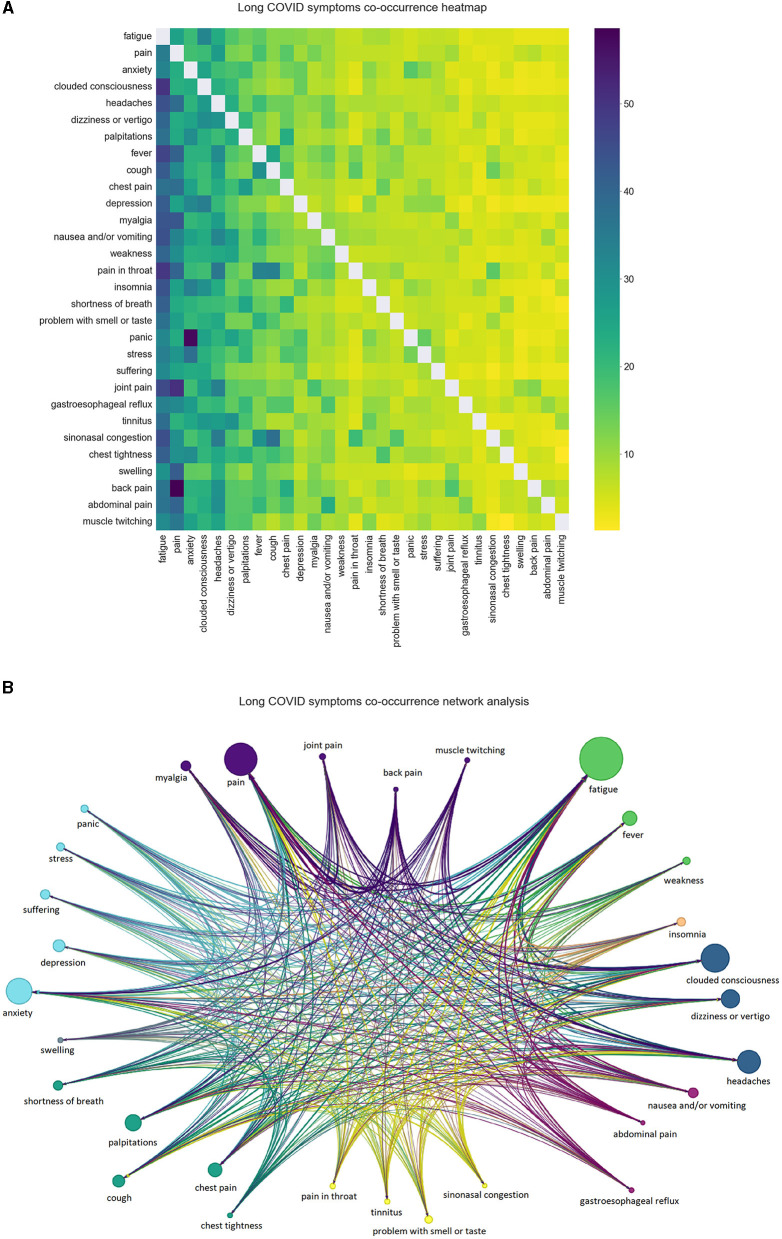
**(A, B)** Long COVID symptoms co-occurrence heatmap and network analysis. The heatmap is a representation of the percentage of the co-occurrence of a symptom (on the y-axis) with another symptom (on the x-axis) out of all its occurrences.

Out of all the processed posts, 78.7% mentioned at least one symptom and 58.42% of all the preprocessed posts contained at least 2 symptoms. The 30 most frequent symptoms across our processed data can be seen in Figure 3. The occurrence reflects the number of posts where the symptom was mentioned at least once. Fatigue, pain, clouded consciousness, anxiety and headaches were all found in more than 12.5% of the posts. We then plotted the co-occurrences of symptoms in Figure 4A. Two symptoms are considered to be co-occurring when they were both mentioned in the same post. For instance, out of the total mentions of depression, around 42% occurred with fatigue, 35% with clouded consciousness and 32% with anxiety. Naturally, the co-occurrence percentage of all symptoms is higher with the most frequent symptoms which explains the darker hues on the left side of the heatmap that become lighter as the symptoms become less frequent. The relationship between the symptoms can be explored further in the network in Figure 4B. The node size reflects the frequency, the directed edges echo the co-occurrence percentage and the color represents the category of the symptoms. There are multiple connections between the different nodes reflecting the high likelihood of experiencing more than one of the frequent symptoms at once. Strong connections mirrored by thick edges are directed toward recurrent symptoms such as fatigue and pain.

**Figure 5:**
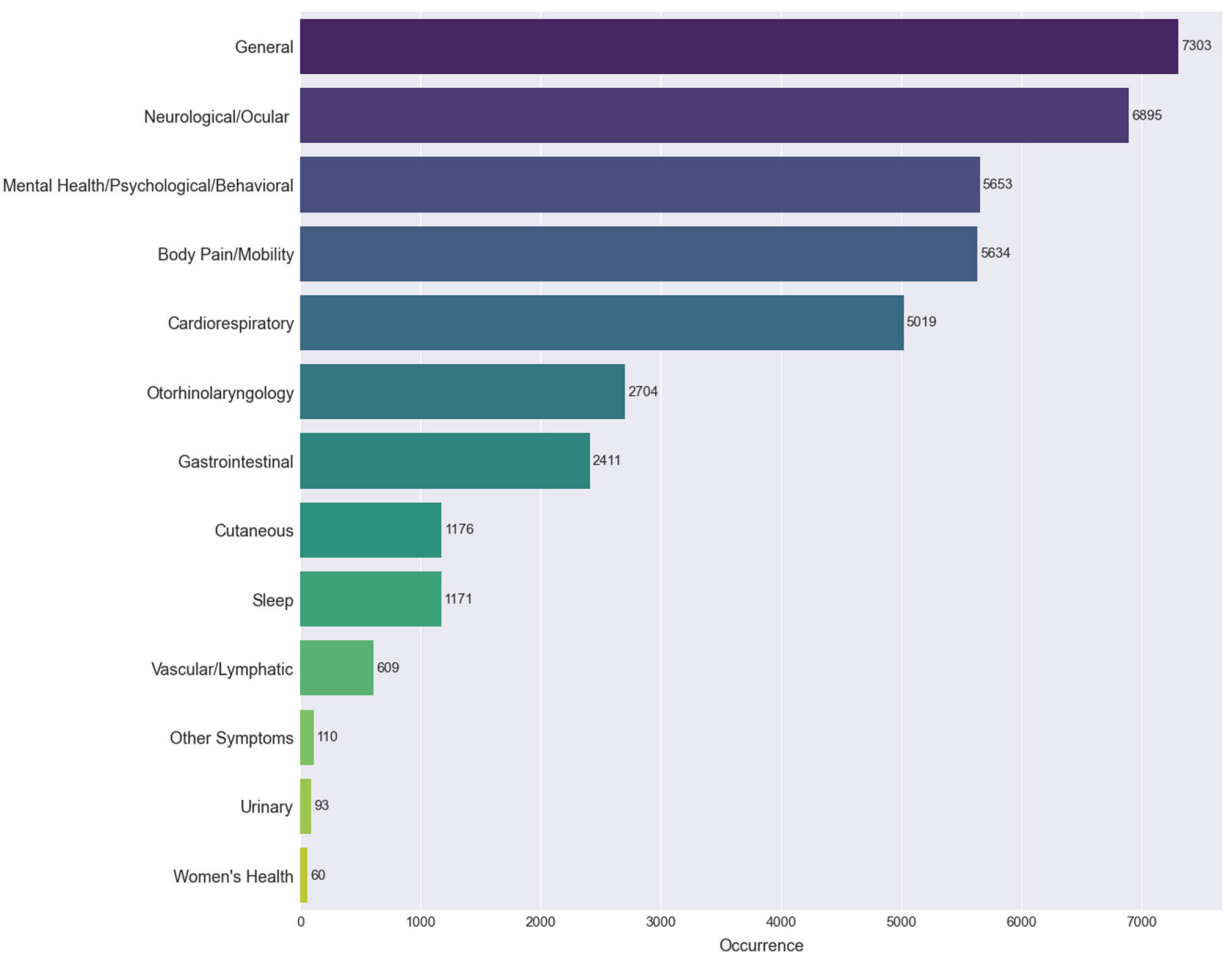
Most frequently extracted categories of Long COVID symptoms. The ranking of the 13 categories of symptoms according to the occurrence of their associated symptoms.

**Figure 6:**
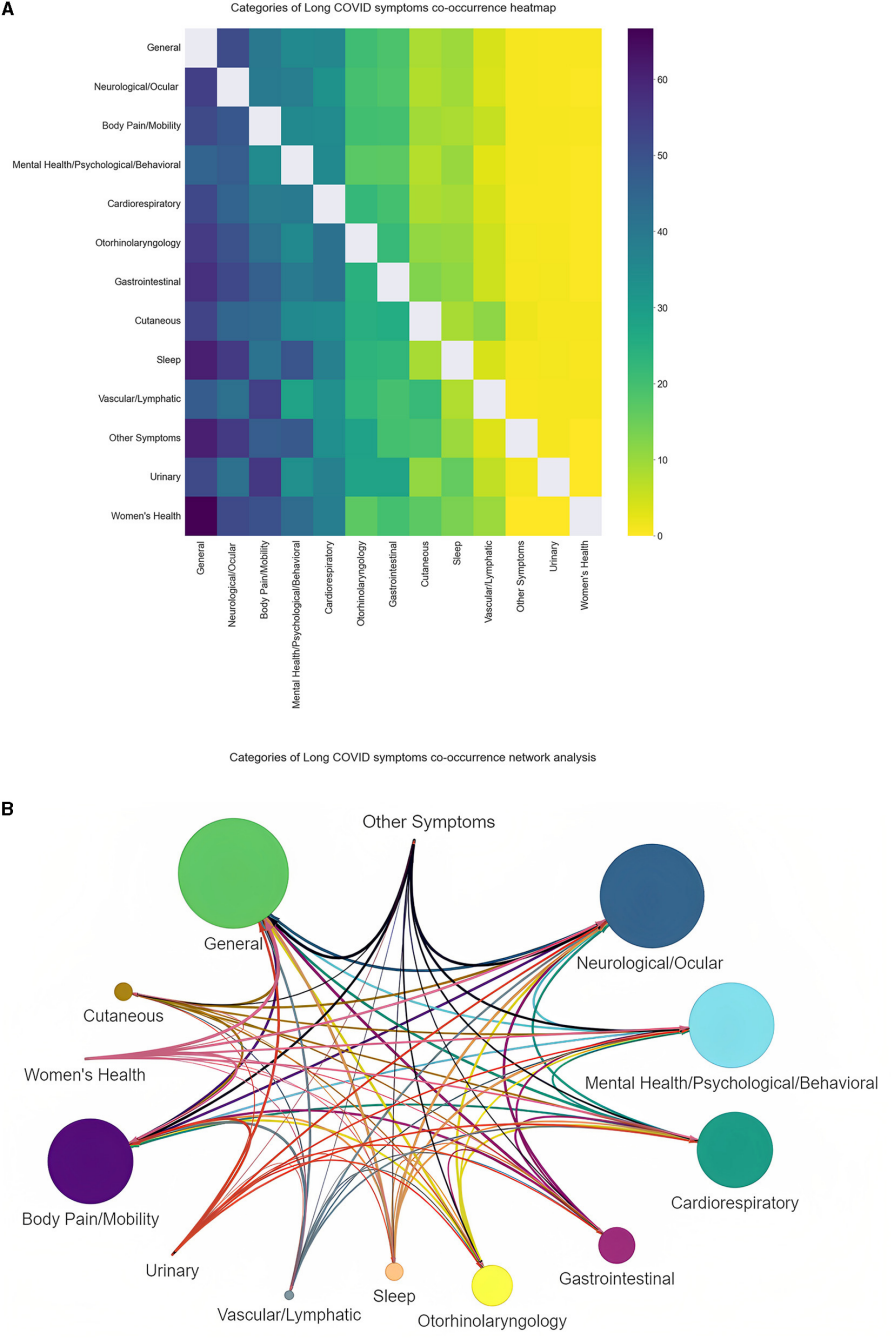
**(A, B)** Categories of Long COVID co-occurrence heatmap and network analysis. The heatmap is a representation of the percentage of the co-occurrence of a category of symptoms (on the y-axis) with another category of symptoms (on the x-axis) out of all its occurrences.

We used the same visualization methods to explore the co-occurrences from a clearer and a more general perspective using the categories of symptoms. In Figure 5, we observe the prevalence of each category of symptoms through the number of posts they appear in. General symptoms (e.g., fatigue and fever) represent the most frequent category with which the remaining categories have high co-occurrence percentages, according to Figure 6. For example, we can see that sleep disorders are strongly connected to general, neurological and ocular, and mental health, psychological and behavioral issues.

### Symptoms clustering

The initial execution of the K-means algorithm on the preprocessed 20,340 posts from 7,560 distinct users returned K = 53 as the optimal number of clusters. However, the following issues were detected for the resulting clusters: a negative silhouette score of −0.00124, 8 clusters out of the 53 only contain a single post that could potentially be included in other clusters, multiple clusters were very similar in terms of the subject. We then explored the clusters for K in (25, 53) and found that for K = 35, we have the highest positive silhouette score of 0.00983 and only one cluster amongst the 35 contained a single outlier post which was ignored. We managed to identify the clusters and their key topics. Twenty-eight clusters mostly focused on a specific symptom, two had mentions of mixed symptoms, three contained symptoms triggered by external factors (e.g., exercising), one cluster was vaccine-related and one cluster grouped recovery and relapse experiences.

More details on separate and group labels for the resulting clusters and the word clouds are respectively in Supplementary material 4, 5.

The biggest cluster was composed of around 28% of the total posts. 60% of the posts in this cluster contained at least one symptom from the PASC lexicon. Many of the posts closest to the cluster center were long descriptions of different Long COVID experiences that mention a variety of symptoms. Fatigue was mentioned in almost 11% of the posts, followed by anxiety (8%) and pain (5%). On a bigger scale, mental health, psychological and behavioral symptoms were the most cited with a percentage of 24% out of all posts and followed by general symptoms (e.g., fatigue, around 21%) then neurological and ocular symptoms (e.g., headaches, 14%).

Three clusters contained posts related to post-exertional malaise, symptoms that would be triggered by physical activities and beverages (e.g., alcohol, coffee). The most frequently mentioned symptoms in these clusters were fatigue (16%), dizziness or vertigo (9.3%), clouded consciousness (6.3%), anxiety (4.5%) and headaches (4.1%).

**Figure 7:**
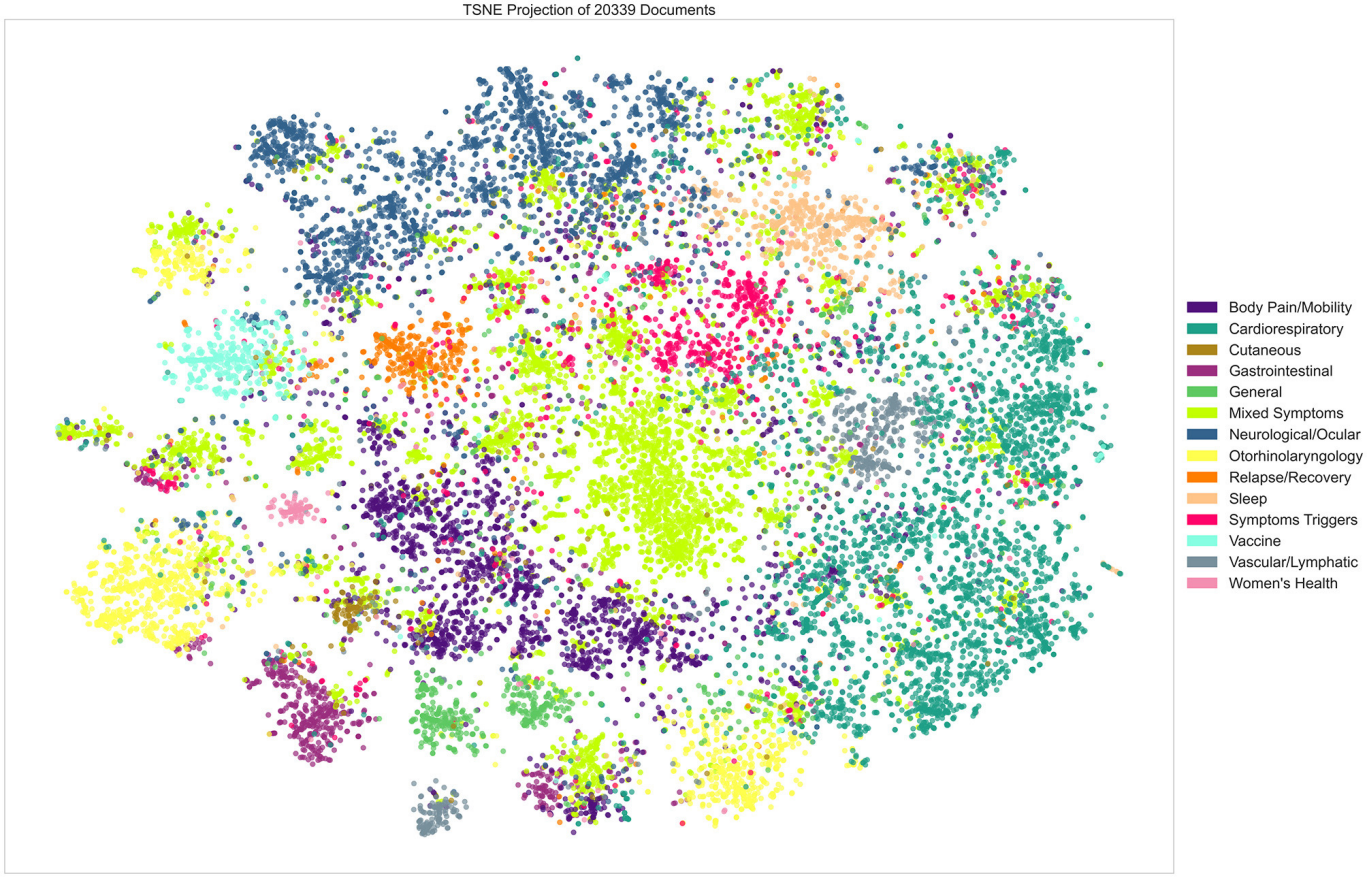
Visual representation of the grouped clusters (t-SNE analysis).

The grouped clusters were projected using the t-SNE algorithm in Figure 7. Visual distinction between several groups and the clusters within the same group is clear despite the presence of some overlapping data points. We chose to only visualize groups of clusters as projecting all of the individual clusters results in a very busy and indistinguishable visualization. We further analyzed those groups by extracting the most frequent symptoms and categories of symptoms within each one. Table 1 shows the results of this extraction and we can notice the prevalence of the general symptoms that range between 13 and 34% of all symptoms found in any of the different labeled groups of clusters. Fatigue is the general symptom which is almost always present. Neurological and Ocular issues such as headaches are also important and are often present with other types of symptoms. Reasonably, mental health, psychological and behavioral problems, despite not showing in the clustering result as a separate cluster, are still prevalent alongside the other types of symptoms and reflect the struggle of the patients no matter the physical manifestation of their Long COVID.

**Table 1:** Distribution of the symptoms and the categories of symptoms in the cluster groups.

**Cluster label**	**Posts with symptoms**	**Top individual symptoms**		**Top categories of symptoms** ^*^	
Vascular/Lymphatic	68.22%	Swelling	9.04%	General	16.68%
		Fatigue	8.32%	Body pain/Mobility	15.08%
		Pain	7.89%	Neurological/Ocular	14.52%
Cardiorespiratory	86.55%	Palpitations	7.61%	Cardiorespiratory	27.24%
		Chest pain	7.19%	General	16.33%
		Fatigue	7.07%	Mental health/Psychological/Behavioral	14.81%
Otorhinolaryngology	83.14%	Smell/taste problems	8.48%	Otorhinolaryngology	26.78%
		Pain in throat	7.39%	General	15.64%
		Fatigue	7.06%	Neurological/Ocular	12.78%
General	91.70%	Fever	13.55%	General	33.76%
		Fatigue	8.88%	Neurological/Ocular	12.83%
		Pain	6.04%	Body pain/Mobility	12.11%
Sleep	85.40%	Insomnia	12.51%	Sleep	20.89%
		Fatigue	11.19%	General	19.55%
		Anxiety	7.27%	Mental health/Psychological/Behavioral	15.53%
Neurological/Ocular	92.69%	Clouded consciousness	18.73%	Neurological/Ocular	37.98%
		Headaches	9.51%	General	17.02%
		Fatigue	9.45%	Mental health/Psychological/Behavioral	14.19%
Body pain/Mobility	95.01%	Pain	12.16%	Body Pain/Mobility	30.1%
		Fatigue	5.75%	General	16.26%
		Headaches	3.74%	Neurological/Ocular	14.04%
Gastrointestinal	77.92%	Fatigue	8.04%	General	22.79%
		Nausea and/or vomiting	5.95%	Gastrointestinal	18.71%
		Pain	4.45%	Neurological/Ocular	13.44%
Cutaneous	91.97%	Hives	17.22%	Cutaneous	41.19%
		Itching	11.96%	General	13.73%
		Rash	10.04%	Mental health/Psychological/Behavioral	9.85%
Women's health	76.83%	Fatigue	9.49%	General	21.94%
		Pain	8.1%	Body pain/Mobility	16.54%
		Headaches	4.46%	Neurological/Ocular	14.74%

^*^Symptoms from the PASC lexicon were classified into 13 categories detailed in Supplementary material 3.

## Discussion

### Long COVID symptomatology

We managed to study the experience of people with Long COVID from their own perspective using Reddit. Text mining techniques such as keyword extraction and Machine Learning algorithms such as classification and clustering were used to better understand the symptomatology and concerns of people with Long COVID.

When it comes to the most frequent symptoms of Long COVID, although prominent symptoms such as pain, fatigue and mental health problems are among the top experienced symptoms across research works, the results in this work slightly differ from previous studies in terms of numbers. Sarker and Ge study on “covidlonghaulers” subreddit posts used the approximate matching method and found that the most frequently reported symptoms were mental health-related (55.2%), fatigue (51.2%), general ache/pain (48.4%), brain fog/confusion (32.8%), and dyspnea (28.9%) (16). While establishing a Long COVID lexicon from health records, Wang et al. highlighted the most common symptoms in data from clinical records (17), which can differ from Reddit data, especially in terms of demographics and language vocabulary. The top symptoms they identified were pain (43.1%), anxiety (25.8%), depression (24.0%), fatigue (23.4%), and joint pain (21.0%). On the other hand, we worked on several Long COVID-related subreddits, and using a lexicon-based keyword extraction, the most common symptoms were fatigue (29.4%), pain (22%), clouded consciousness (19.1%), anxiety (17.7%) and headaches (15.6%). The top categories of symptoms were General (45.5%), Neurological/Ocular (42.9%), Mental Health/Psychological/Behavioral (35.2%), Body Pain/Mobility (35.1%) and Cardiorespiratory (31.2%). The differences with the previously cited work on Reddit (16) could be explained by the different text analysis methods used for the identification of the symptoms as well as the difference in the data sample size affected by the source subreddits, time period of the collection and the inclusion or exclusion of the comments in the analyzed data. Adding to those differences, our analysis of the data was also expanded by measuring the associations between the symptoms through co-occurrence heatmaps, network analysis and clustering. The clustering also helped automatically match posts that did not mention specific symptoms from the lexicon with others discussing the same topics.

Zhang et al. worked on the identification of PASC topics and subphenotypes using cohorts data from the National Patient-Centered Clinical Research Network (18). They identified in the first place ten distinct topics describing each a set of co-occurring PASC diagnoses. Understandably, symptoms of the same disease category are often mentioned in the same topic, such as diseases of the digestive system and diseases of the musculoskeletal system and connective tissue. Breathing abnormality and throat/chest pain, abdominal and pelvic pain, headache, malaise and fatigue were mentioned in three or more topics. Our results show that pain in general, fatigue and headaches, three of the most frequent symptoms, also have important co-occurrence scores with various other symptoms. Furthermore, the cited work defines four PASC subphenotypes that could be compared to the co-occurrences between the categories of symptoms found in our data. Notably, the body pain and mobility symptom category is mentioned frequently with the neurological and ocular symptom category (around 40% of the time neurological and ocular symptoms were mentioned, they were associated with body pain and mobility issues) which can potentially fall under Subphenotype 3 (musculoskeletal and nervous).

We were able to display the relationship between the various spontaneously reported symptoms of Long COVID and how a variety of them could be experienced at once. High co-occurrence rates were found between fatigue, pain, clouded consciousness, headaches and anxiety. General and Neurological/Ocular symptoms were found important in the different groups of symptoms in our clustering results. In addition, the data used for this research project covered a two-year period which was enriching in the case of a recent condition such as Long COVID. Important clusters related to vaccines and recovery/relapse experiences were also identified.

As discussed in the previous paragraphs, we found similarities with results in research based on clinical records data. This shows how the shared self-reported outcomes from patients all around the world, who probably feel more at ease expressing themselves anonymously to a community with similar interests (26, 27), could support clinical and epidemiological studies performed on other populations of a smaller scale or a different demographic. It could also help orient clinical research according to the most frequently discussed issues. Generally speaking, this work also proves the complementary value of social media data in health research.

This study has also several limitations. First, text in social media discourse does not necessarily stick to language rules such as grammar and can be informal with extended use of abbreviations (e.g., using acronyms and internet slang) (28). It is possible that the applied text normalization steps were not exhaustive and did not take into consideration all of those exceptions. In the same context, the usage of a lexicon extracted from clinical records on our input text cannot guarantee the inclusion of all of the symptom vocabulary used in more than 20,000 Reddit posts. Rarely mentioned symptoms that were not part of the lexicon and that did not appear in the most frequent words, could have been overlooked. Furthermore, the classification of the lexicon symptoms in categories was manually done, which could have led to potential misclassifications. Finally, the choice of the number of clusters K after multiple iterations and the manual labeling process do not completely eliminate the possibility of having outliers and non-completely homogenous clusters when it comes to the topic of interest (29).

### Perspectives

The data collection could be extended to other social media platforms, especially Twitter, the social media where the concept of Long COVID was first depicted and where the first experiences with the condition were shared. This would provide a more inclusive overview in terms of the number and variety of the experiences of the disease itself and the demographic characteristics of the patients such as gender (30, 31).

Future analysis of how the patient-reported symptoms evolve over time will improve our understanding of the manifestation of Long COVID. Other important concerns of the community such as relapse, recovery, the ability to return to work, the age groups of patients and vaccination are also important to explore in follow-up research works.

## Conclusion

We have shown that social media is a valuable source of information that helps to orient research in public and digital health. This study gives a thorough analysis of Long COVID symptomatology based on the patient-reported outcomes on Reddit. General symptoms, mainly fatigue, were found to be the most prominent since 2020. Co-occurrence scores between the different categories of symptoms and our network analysis proved that people with Long COVID can experience a wide range of symptoms simultaneously and that the symptom profiles of patients are heterogeneous. Other concerns such as vaccination and relapse after recovery were also put into perspective.

## Data availability statement

The data analyzed in this study is subject to the following licenses/restrictions: The raw data supporting the conclusions of this article will be made available by the authors, without undue reservation. Requests to access these datasets should be directed to guy.fagherazzi@lih.lu.

## Author contributions

GF takes full responsibility for the work as a whole, for the decision to submit, publish the manuscript, and designed the research. HA, CB, and GF conducted the research and drafted the article. HA and CB collected, labeled, analyzed, and interpreted the data. AF and MG revised the manuscript critically. All authors contributed to the article and approved the submitted version.

## Supplementary material

Supplementary Material 1 Members and posts count across subreddits.

Supplementary Material 2 Personal Long COVID text classifier performance.

Supplementary Material 3 Categorization of PASC lexicon symptoms.

Supplementary Material 4 Clusters description.

Supplementary Material 5 Word cloud representation of the clusters.
