# Supplementary material for: The Long COVID experience from a patient's perspective: a clustering analysis of 27,216 Reddit posts
Source: Front Public Health. 2023 Aug 17;11:1227807. doi: 10.3389/fpubh.2023.1227807 (PMC10470115; doi:10.3389/fpubh.2023.1227807)
Supplement: Supplementary Material 5 — Word cloud representation of the clusters. [file Image_1.PDF]

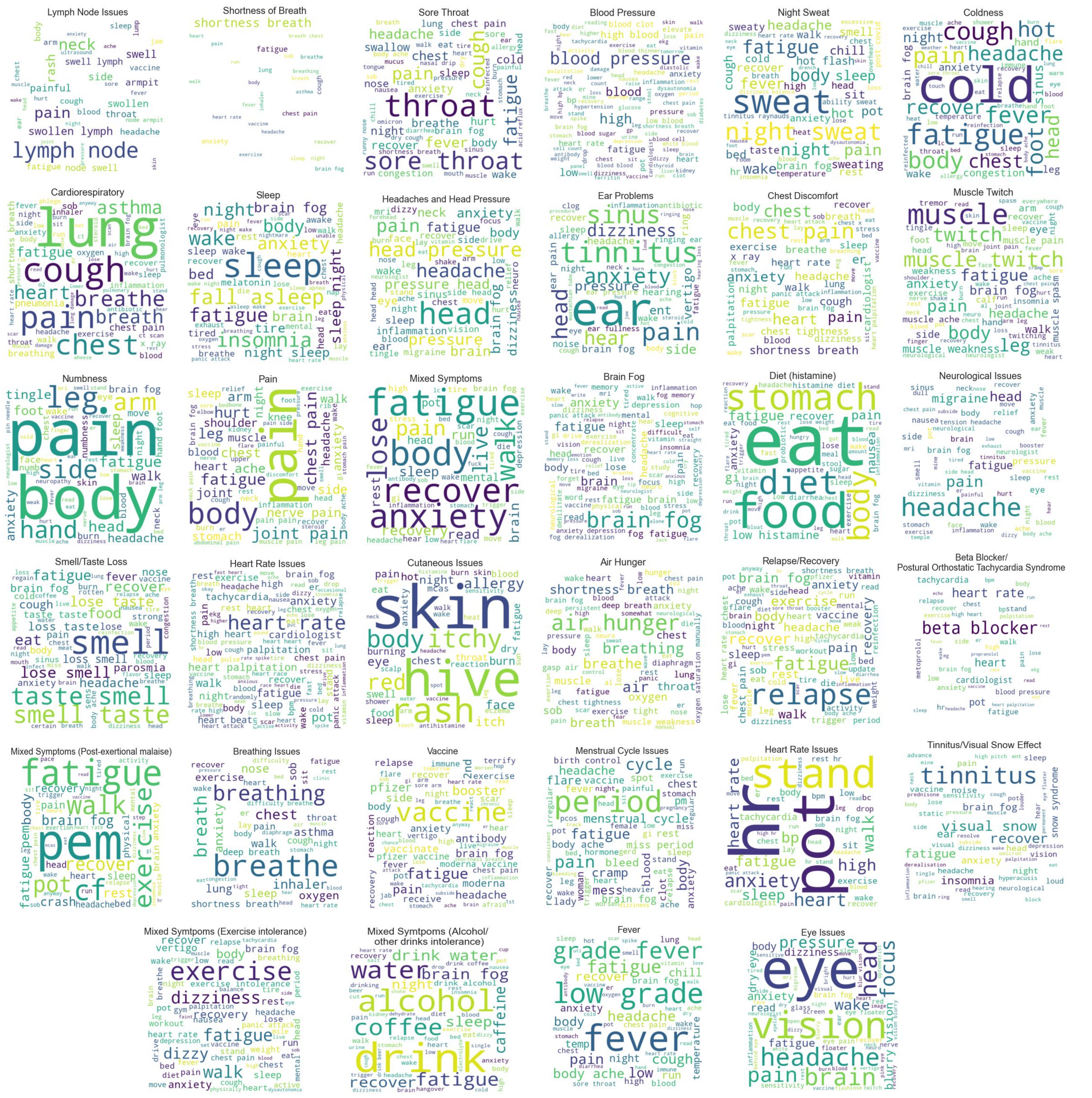
Lymph Node Issues
Shortness of Breath
Sore Throat
Blood Pressure
Night Sweat
Coldness
Cardiorespiratory
Sleep
Headaches and Head Pressure
Ear Problems
Chest Discomfort
Muscle Twitch
Numbness
Pain
Mixed Symptoms
Brain Fog
Diet (histamine)
Neurological Issues
Smell/Taste Loss
Heart Rate Issues
Cutaneous Issues
Air Hunger
Relapse/Recovery
Beta Blocker/
Postural Orthostatic Tachycardia Syndrome
Mixed Symptoms (Post-exertional malaise)
Breathing Issues
Vaccine
Menstrual Cycle Issues
Heart Rate Issues
Tinnitus/Visual Snow Effect
Mixed Symtpoms (Exercise intolerance)
Mixed Symtpoms (Alcohol/
other drinks intolerance)
Fever
Eye Issues
